# Supplementary material for: Determination of the affinity constants for phage display albumin-binding peptides
Source: PeerJ. 2023 May 22;11:e15078. doi: 10.7717/peerj.15078 (PMC10215749; doi:10.7717/peerj.15078)
Supplement: Supplemental Information 1 [file peerj-11-15078-s001.docx]

Table 4. HSA-3 phage relative affinity constant measurement (for Figure1.)

| Sample | Input phage titer  Φ_T_  （pfu/L） | Input phage concentration  [Φ]_T_  （mol/L） | Elute phage titer  Φ_e_  （pfu/L） | Elute phage concentration  [Φ]_e_  （mol/L） |
| --- | --- | --- | --- | --- |
| 1 | 1.13×10^14^ | 5.32×10^6^ | 7.94×10^6^ | 7.58×10^-13^ |
| 2 | 5.8×10^13^ | 1.03×10^7^ | 7.5×10^6^ | 7.98×10^-13^ |
| 3 | 3.36×10^13^ | 1.79×10^7^ | 5.68×10^6^ | 1.06×10^-14^ |
| 4 | 2.16×10^13^ | 2.78×10^7^ | 2.52×10^6^ | 2.39×10^-14^ |
| 5 | 1.52×10^13^ | 3.99×10^7^ | 2×10^6^ | 3.03×10^-14^ |

Table 5. HSA-4 phage relative affinity constant measurement (for Figure 2.)

| 样品 | Input phage titer  Φ_T_  （pfu/L） | Input phage concentration  [Φ]_T_  （mol/L） | Elute phage titer  Φ_e_  （pfu/L） | Elute phage concentration  [Φ]_e_  （mol/L） |
| --- | --- | --- | --- | --- |
| 1 | 5.90×10^13^ | 1.02×10^7^ | 5.0×10^6^ | 1.2×10^-14^ |
| 2 | 3.88×10^13^ | 1.55×10^7^ | 4.32×10^6^ | 1.4×10^-14^ |
| 3 | 2.93×10^13^ | 2.05×10^7^ | 2.87×10^6^ | 2.1×10^-14^ |
| 4 | 2.48×10^13^ | 2.44×10^7^ | 2.8×10^6^ | 2.2×10^-14^ |
| 5 | 2.10×10^13^ | 2.86×10^7^ | 2.16×10^6^ | 2.8×10^-14^ |
