# Supplementary material for: Determination of the affinity constants for phage display albumin-binding peptides
Source: PeerJ. 2023 May 22;11:e15078. doi: 10.7717/peerj.15078 (PMC10215749; doi:10.7717/peerj.15078)
Supplement: Supplemental Information 2 [file peerj-11-15078-s002.pdf]

Primers used for sequencing phage display binding peptide

PIII leader sequence *Kpn* I

5'···TTA TTC GCA ATT CCT TTA GTG GTA CCT TTC TAT TCT CAC TCT

3'···AAT AAG CGT TAA GGA AAT CAC CAT GGA AAG ATA AGA GTG AGA

···Leu Phe Ala Ile Pro Leu Val Val Pro Phe Tyr Ser His Ser

↓ Start of mature heptapeptide-gIII fusion ***Eag* I**

NNK NNK NNK NNK NNK NNK NNK GGT GGA GGT TCG GCC GAA ACT GTT GAA

NNM NNM NNM NNM NNM NNM NNM CCA CCT CCA AGC CGG CTT TGA CAA CTT

Xxx Xxx Xxx Xxx Xxx Xxx Xxx Gly Gly Gly *Ser Ala Glu Thr Val Glu*

AGT TGT TTA GCA AAA TCC CAT ACA GAA AAT TCA TTT ACT AAC GTC TGG

TCA ACA AAT CGT TTT AGG GTA TGT CTT TTA AGT AAA TGA TTG CAG ACC

Ser Cys Leu Ala Lys Ser His Thr Glu Asn Ser Phe Thr Asn Val Trp

←-28 gIII Primer

AAA GAC GAC AAA ACT TTA GAT CGT TAC GCT AAC TAT GAG GGC···–3'

TTT CTG CTG TTT TGA AAT CTA GCA ATG CGA TTG ATA CTC CCG···–5'

Cys Asp Asp Lys Thr Leu Asp Arg Tyr Ala Asn Tyr Glu Gly···

←-96 gIII Primer

K-G or T;M-A or C

Figure 3. The N-terminal sequence of seven random peptide-gIII fusion protein

Sequencing HSA-3 binder Result

CTTTCGGACGTTAGTAAATGAATTTTCTGTATGGGATTTTGCTAAACAAC
TTTCAACAGTTTCGGCCGA**ACCTCCACC****ATTAAGCTTCTAACTCTCCGC**A
GAGTGAGAATAGAAAGGTACCACTAAAGGAATTGCGAATAATAATTTTTT
CACGTTGAAAATCTCCAAAAAAAAAGGCTCCAAAAGGAGCCTTTAATTGTA
TCGGTTTATCAGCTTGCTTTCGAGGTGAATTTCTTAAACAGCTTGATACC
GATAGTTGCGCCGACAATGACAACAACCATCGCCCACGCATAACCGATAT
ATTCGGTCGCTGAGGCTTGCAGGGAGTTAAAGGCCGCTTTTGCGGGATCG
TCACCCTCAGCAGCGAAAGACAGCATCGGAACGAGGGTAGCAACGGCTAC
AGAGGCTTTGAGGACTAAAGACTTTTTCATGAGGAAGTTTCCATTAAACG
GGTAAAATACGTAATGCCACTACGAAGGCACCAACCTAAAACGAAAGAGG
CAAAAGAATACACTAAAACACTCATCTTTGACCCCCAGCGATTATACCAA
GCGCGAAACAAAGTACAACGGAGATTTGTATCATCGCCTGATAAATTGTG
TCGAAATCCGCGACCTGCTCCATGTTACTTAGCCGGAACGAGGCGCAGAC
GGTCAATCATAAGGGAACCGAACTGACCAACTTTGAAAGAGGACAGATGA
ACGGTGTACAGACCAGGCGCATAGGCTGGCTGACCTTCATCAAGAGTAAT
CTTGACAAGAACCGGATATTCATTACCCAAATCAACGTAACAAAGCTGCT
CATTCAGTGAATAAGGCTTGC

GenBank Submissions # 2410423

-96 gIII Primer sequencing: 5’-ATTAAGCTTCTAACTCTCCGC-3’

HSA-3 DNA: 5’-GCG GAG AGT TAG AAG CTT AAT-3’

HSA-3 peptide: Ala-Glu-Ser-Gln-Lys-Leu-Asn (AESQKLN)

Sequencing HSA-4 binder Result

CAGACGTTAGTAAATGAATTTTCTGTATGGGATTTTGCTAAACAAC
TTTCAACAGTTTCGGCCGA**ACCTCCACCACGCAACGGAAGCGGCGTAAG**A
GAGTGAGAATAGAAAGGTACCACTAAAGGAATTGCGAATAATAATTTTTT
CACGTTGAAAATCTCCAAAAAAAAAGGCTCCAAAAGGAGCCTTTAATTGTA
TCGGTTTATCAGCTTGCTTTCGAGGTGAATTTCTTAAACAGCTTGATACC
GATAGTTGCGCCGACAATGACAACAACCATCGCCCACGCATAACCGATAT
ATTCGGTCGCTGAGGCTTGCAGGGAGTTAAAGGCCGCTTTTGCGGGATCG
TCACCCTCAGCAGCGAAAGACAGCATCGGAACGAGGGTAGCAACGGCTAC
AGAGGCTTTGAGGACTAAAGACTTTTTCATGAGGAAGTTTCCATTAAACG
GGTAAAATACGTAATGCCACTACGAAGGCACCAACCTAAAACGAAAGAGG
CAAAAGAATACACTAAAACACTCATCTTTGACCCCCAGCGATTATACCAA
GCGCGAAACAAAGTACAACGGAGATTTGTATCATCGCCTGATAAATTGTG
TCGAAATCCGCGACCTGCTCCATGTTACTTAGCCGGAACGAGGCGCAGAC
GGTCAATCATAAGGGAACCGAACTGACCAACTTTGAAAGAGGACAGATGA
ACGGTGTACAGACCAGGCGCATAGGCTGGCTGACCTTCATCAAGAGTAAT
CTTGACAAGAACCGGATATTCATTACCCAAATCAACGTAACAAAGCTGCT
CATTCAGTGAATAAGGCTTGC

Genebank Submission # 2411052

-96 gIII Primer sequencing： 5’ ACGCAACGGAAGCGGCGTAAG 3’

HSA-4 DNA: 5’-CTT ACG CCG CTT CCG TTG CGT-3’

HSA-4 peptide： Leu-Thr-Pro-Leu-Pro-Leu-Arg （ITPLPLR）
